# Using an agent-based model to simulate children’s active travel to school

**DOI:** 10.1186/1479-5868-10-67

**Published:** 2013-05-26

**Authors:** Yong Yang, Ana V Diez-Roux

**Affiliations:** 1 Department of Epidemiology, University of Michigan, 1415 Washington Heights, Ann Arbor, Michigan 48109, USA

## Abstract

**Background:**

Despite the multiple advantages of active travel to school, only a small percentage of US children and adolescents walk or bicycle to school. Intervention studies are in a relatively early stage and evidence of their effectiveness over long periods is limited. The purpose of this study was to illustrate the utility of agent-based models in exploring how various policies may influence children’s active travel to school.

**Methods:**

An agent-based model was developed to simulate children’s school travel behavior within a hypothetical city. The model was used to explore the plausible implications of policies targeting two established barriers to active school travel: long distance to school and traffic safety. The percent of children who walk to school was compared for various scenarios.

**Results:**

To maximize the percent of children who walk to school the school locations should be evenly distributed over space and children should be assigned to the closest school. In the case of interventions to improve traffic safety, targeting a smaller area around the school with greater intensity may be more effective than targeting a larger area with less intensity.

**Conclusions:**

Despite the challenges they present, agent based models are a useful complement to other analytical strategies in studying the plausible impact of various policies on active travel to school.

## Background

Active travel to school (ATS) constitutes a substantial portion of children’s overall physical activity [1] and ATS is associated with higher overall physical activity [2-10]. ATS can decrease traffic and pollution, reduce children’s dependence on parents, improve social interactions, and promote healthy life styles which may be maintained into adulthood [11]. Despite the multiple merits associated with ATS, today fewer than 15% US children and adolescents walk or bicycle to school, compared to about 50% in 1969 [12]. Recently, a number of intervention programs [13-15] have been applied to encourage ATS. However, intervention studies are in a relatively early stage and evidence of their effectiveness over long periods is limited [16].

Research has identified a number of factors that are associated with ATS including the characteristics of children and families [5,10,17,18] (such as age and fitness of the child, neighbourhood safety and traffic safety, and household transportation options), features of schools [5,10,19,20] (such as distance from the household, and school bus policy), and features of neighbourhoods [5,10,17,21,22] (such as urban form and social norms). Generally, boys, Hispanic and African American children, children from lower SES families, and children attending public schools, living close to schools, and living in neighbourhoods with walking or bicycling friendly environments are more like to actively travel to school [23].

Long distance to school is the most common barrier to ATS among US children [24]. Distance to school is a function of the number of schools, their location, and the definition of catchment areas. A longer distance to school has been found to be associated with lower ATS, especially when the distance is less than one mile [25,26]. It has been estimated that 47% of the decline in walking to school that occurred between 1969 and 2001 is attributable to increases in the distances between households and schools [27] resulting at least in part from suburbanization and from the creation of larger school districts. Communities across the USA have been shifting away from small, neighbourhood schools to larger schools located in areas where housing densities are low and road networks lack connectivity [28]. Studies [29-31] have reported that high population density, and small enrollment size are associated with higher percentage of ATS. However, understanding of how the definition of school catchment areas affects ATS is limited. It is also unclear how school catchment area definition, school size, and population density may jointly affect ATS.

Traffic-related danger is another major barrier to ATS [24]. However evidence of the impact of changing traffic safety levels is mixed [10,32]. The Safe Routes to School [14] program is a nationwide initiative to improve traffic safety. One way to increase traffic safety is to modify road conditions such as creating crossings, improving sidewalks, and implementing traffic calming measures. An important decision is where interventions should be targeted in order to get the maximum benefit with limited resources, for example, should the investment focus on a more intense intervention on a smaller area, or a less intense intervention on a bigger area?

The majority of research on factors related to children’s school commuting has relied on statistical models applied to observational data. Most analyses have been cross-sectional, have investigated only a limited number of characteristics, and have not fully explored interactions between factors. In general, existing work fails to capture the dynamic interactions between individuals (e.g. the effects of social networks) or the dynamic interactions between individual and environmental factors (e.g. feedback loops between the walking behavior of individuals and the safety of an area). In addition, features of built and social environments may dynamically interact (e.g. mixed land use and land use density may affect levels of safety). Existing research may provide an incomplete basis for policy because of its inability to draw inferences regarding the plausible impact of interventions in the context of dynamic systems.

Agent-based modeling (ABM) has been increasingly applied to population health problems [33,34] because of its utility in studying numerous agents specified at various scales, capturing the complex dynamic relationships, and accounting for feedbacks between individuals and between individuals and their environments. By focusing on explicit processes and considering how the outcomes of the functioning of the systems would change if features were modified, ABMs can be used to conduct various “what-if” experiments relevant to policy or intervention [35]. To our knowledge, applications of ABMs to the study of how the social and built environments shape people’s active travel behavior remains limited [36-38], and has not yet focused on children.

We developed an ABM to simulate children’s school travel behavior within a hypothetical city. The model was calibrated to existing data. We illustrate the utility of this approach by applying the model to two outstanding questions in ATS: (1) the impact of school location, catchment area definition, school size, and population density; and (2) the impact of changing traffic safety levels.

## Methods

### Model

The model was developed in Java and Repast [39]. It includes a hypothetical city with a road network, schools, households and children. The city has a size of 8 km × 8 km, and has a grid road network with 41 vertical and 41 horizontal roads and evenly sized blocks. Each road has 40 segments separated by road intersections. Each segment (composed of 10 cells) denotes 200 m in reality. Each cell (denoting a location with the size of 20 m × 20 m in reality) can be occupied by a household, a school or be empty. The city includes four middle schools and 3000 households. Households are randomly distributed across the city. Each household includes one child and the child is enrolled in one of the four middle schools. Each household has a level of concern towards traffic safety (denoted by *C*_*t*_) with respect to children walking to school. *C*_*t*_ is a threshold value: if traffic safety is perceived to be above that value, the household may allow the child to walk to school. Each child has an attitude towards walking (denoted by *A*_*w*_): a higher value of *A*_*w*_ indicates a higher probability of walking to school (assuming other conditions are met, as discussed below). Both *C*_*t,*_ and *A*_*w*_ are assigned at model initialization using a random draw from a uniform distribution between 0 and 1. For the purposes of this illustration, *C*_*t,*_ remains constant but *A*_*w*_ is updated over time based on various feedbacks. The model is time-discrete with each step being one day.

Each day, children travel to school by walking or are driven by their parents. Because distance to school and traffic safety are the two most important factors influencing whether children walk to school [24], they are explicitly implemented in the children’s travel mode selection process. It is assumed that a child will walk to school if the safety along the walking route is above the household’s concern towards traffic safety and if the child’s attitude towards walking is high and/or the distance to school is short. Thus, a child will travel to school by walking if both conditions below are met:

(1) $$S_{t}>C_{t}$$

(2) $$A_{w}+P_{d}>1$$

Where *S*_*t*_ is the mean traffic safety of all the cells along the route from household to school, *C*_*t*_ is the household’s concern towards traffic safety, *A*_*w*_ is the child’s attitude towards walking, and *P*_*d*_ is the probability of walking given the distance to the school. *P*_*d*_ is computed as follows:

(3) $$P_{d}=e^{-\mathrm{\beta d}}$$

Where d is the distance from household to school and *β* is the distance decay parameter. The distance decay parameter was calibrated by comparing model output to empirical distributions as described below. Condition 2 implies that the child may walk to school if the child’s attitude towards walking is high and/or if the distance to the school is short (resulting in a higher *P*_*d*_). The use of the sum allows us to account for the fact that children with a high attitude towards walking may walk even if the distance to school is long, and vice versa, when the distance is very short even children with a low attitude may walk to school.

For a given child, the selected travel mode may differ from day to day because although the distance and route from the household to the school and *C*_*t*_ are assumed to be constant over time, both *S*_*t*_ and *A*_*w*_ are updated daily. Each day the traffic safety at each cell is updated as a function of the total number of people who walk by that cell as follows:

(4) $$S_{t}=1-W^{-0.6}$$

Where *W* is the total number of people who walk by that cell each day, which includes the children walking to school and the background walkers (the number of background walkers is assumed to be constant across the city). Prior research has shown that *W*^-0.6^ is a reasonable estimate of the probability that a pedestrian is struck by a car, given *W* walkers in the area [40]. We therefore use 1-*W*^-0.6^ as a measure of traffic safety.

The child’s attitude towards walking is influenced by school social norms regarding walking. Each day the attitude is updated as a function of the total number of children who walk to school as follows:

(5) $$A_{w}^{\prime }=A_{w}\frac{0.9+0.1\frac{N_{w}^{\prime }}{N}}{0.9+0.1\frac{N_{w}}{N}}$$

Where $A_{w}^{\prime }$ and *A*_*w*_ are the attitudes towards walking today and yesterday, respectively. *N* is the total number of children enrolled in the school, and $N_{w}^{\prime }$ and *N*_*w*_ are the total number of children enrolled in the school who travelled to school by walking today and yesterday, respectively. According to this formula, if within a given school, more children walk to school today than yesterday, then the attitudes towards walking of all the children within the school will increase.

To investigate our research questions, we contrasted the percent of children who walk to school under various scenarios. For each scenario the model was run until the number of children who walk to school remains constant (approximately 5–10 days). For each scenario, we report the mean percentage of children walking to school and the distribution of children by the distance from their households to school over 20 simulations.

The number of background walkers and β (the distance decay parameter) were jointly calibrated through an iterative process by which model output for the baseline scenario (see below) was compared to data on the percentages of children who walk to school across various categories of the distance to school derived from 2009 National Household Travel Survey (see Table 1). The calibrated values were 1.6 for the distance decay parameter and 1.5 walkers per cell each day for the background walkers.

**Table 1:** Percent of children who walk to school, by distance, from model predictions and 2009 NHTS data

	***Summary statistics generated by the model (baseline scenario, with 20 simulations)***		***Values from NHTS data***[12]	
***Distance (miles)***	***Mean percent***	***Percent range***	***Elementary school***	***Middle school***
<0.25	64.3	50.0-75.4	53.1	65.5
0.25-0.5	38.2	33.5-43.6	25.5	49.9
0.5-1	16.6	14.9-18.4	13.9	18.5
1-2	5.4	4.5-6.6	2.6	7.2
> = 2	1.2	0.4-3.3	1.3	2.0

**Figure 1:**
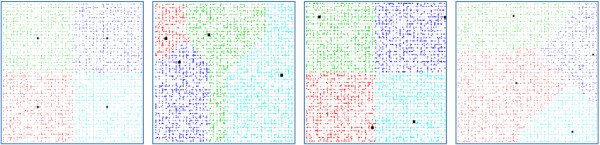
**Schematic display of the four scenarios: Baseline, S1, S2, and S3 (left to right).** Grey lines are roads, black squares are schools, and colored dots are households. Children living in households of the same color attend the same school.

In the baseline scenario, the city is divided into four equal-sized school zones. The four schools are located at the center of each zone as shown in Figure 1. Children are enrolled in the school within their zone. Several scenarios were developed in order to investigate (1) the impact of school location and the definition of catchment areas (which can be modified for example through policies regarding school location and school catchment areas) as well as school size and population density and (2) the impact of changing the traffic safety levels (through for example implementation of traffic calming strategies).

### The impact of school location, catchment area definition, school size and population density

First, to explore the impact of various school location and catchment area policies on ATS, we contrasted the baseline scenario with three scenarios that vary in school location and catchment area. These scenarios are defined in Table 2 and illustrated in Figure 1. Second, we explored the impact of school size (by contrasting scenarios with a fixed number of households but varying numbers of schools, i.e., 2, 4, 6, and 8 schools) and population density (by contrasting scenarios with 1500, 3000, 4500 and 6000 total households). The schools were distributed evenly in the city. When the total number of schools were 2, 4, or 6, the city was divided evenly into 2 × 1, 2 × 2 (the baseline scenario), or 2 × 3 zones, respectively, and the schools were located in the center of each zone. When the total number of schools was 8, the city was divided into 3 × 3 zones, and schools were located in the center of each zone except the zone in the city center.

**Table 2:** Features and simulated results for four scenarios with different school location and catchment area policies

***Scenario***	***Scenario features***		***Simulated results***					
	***School location***	***Definition of catchment area***	***Children walking to school (%)***	***Percent distribution of children by distance to school (in miles)***				
				***<0.25***	***0.25-0.5***	***0.5-1***	***1-2***	***> = 2***
Baseline (B)	Evenly distributed	Children attend nearest school	10.9	2.2	5.8	23.9	59.5	8.7
S1	Randomly located across the whole city	Same as baseline	8.4	2.0	5.1	17.8	42.9	32.3
S2	The city is divided to four school zones of equal size, and the school is randomly located within the zone	Attend school within the same zone	8.2	1.9	4.9	15.7	40.9	36.6
S3	Same as S2	Same as baseline	9.5	2.0	5.6	19.0	49.3	24.3

### Changing the traffic safety level

To investigate this question we used our model to contrast three strategies: (1) increase the safety of all roads within 0.5 miles from the school with value of ***a***; (2) increase the safety of all roads within 0.71 $\left(\approx \frac{\sqrt{2}}{2}\right)$ miles from the school with value of ***a/2*** [we use ***a/2*** because the size of area within 0.71 miles is twice the size of the area within 0.5 miles]; and (3) increase the safety of all roads within one mile from the school with value of ***a/4*** [we use ***a/4*** because the size of area within one mile is four times the size of area within 0.5 miles]. We adjust the intensity of the intervention so that the intensity-area product is equivalent across scenarios (the intervention per unit area is more intense when a smaller area is targeted than when a larger area is targeted). In order to investigate effects across a range of values, three levels of ***a*** (0.1, 0.2 and 0.3) were examined. Because the value of the safety ranges between 0 and 1 by definition, these three values cover a reasonable range of intensity of the intervention.

## Results

### The impact of school location, catchment area definition, school size and population density

Table 2 shows the four scenarios that vary on school location and definition of catchment areas. The baseline scenario has the highest percentage of children walking to school. The percent of children walking to school is higher in scenario S3 than in both scenarios S1 and S2, and walking levels in scenario S1 are very similar to S2. Table 3 shows the percent of children walking to school for scenarios that vary in the total number of schools (i.e., in school size) and in the total number of households (i.e., in population density). Within each row (constant number of households or density) scenarios with more schools (i.e., with smaller catchment areas) have a higher percent of children walking to school. Within each column (constant number of schools) the percent of children walking to school decreases as the number of households (population density) decreases. Diagonally, holding constant the ratio of students per school (1500 households with 2 schools, 3000 households with 4 schools, …), the higher the population density, the higher percent of children walking to school.

**Table 3:** Simulated percent of children walking to school for scenarios with different numbers of schools and households in the city

	***Number of schools***			
***Number of households***	*2*	*4*	*6*	*8*
*6000*	7.5	13.2	16.9	20.0
*4500*	7.0	12.3	15.9	18.7
*3000*	6.3	10.9	14.1	16.8
*1500*	4.8	8.2	10.9	12.6

### Changing the traffic safety level

**Figure 2:**
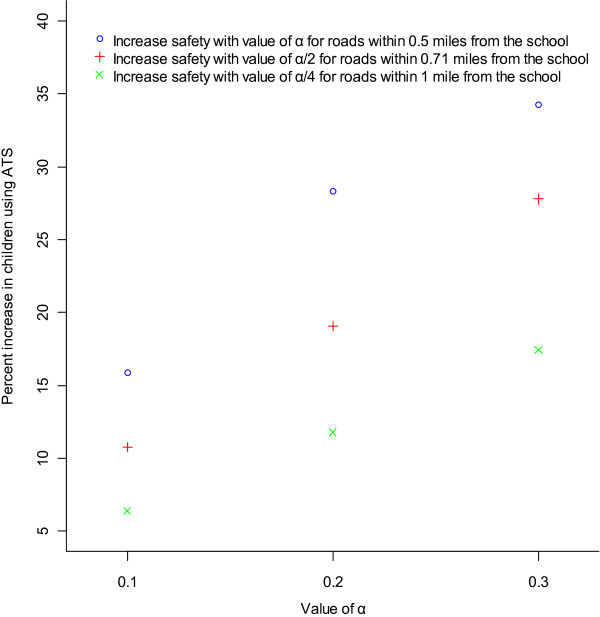
**Percent increase in children using ATS associated with different types of safety interventions (shown by different colors of points) for different intensities (α) of the intervention.**

Figure 2 shows the percent increase in children using ATS associated with improvements in traffic safety for various scenarios. As expected, the increase in the percent of children using ATS is greater as the intensity of the intervention (reflected in the value of ***a***) increases. However, strategies that target a smaller area around the school with greater intensity are more effective in increasing ATS than strategies that target a broader area with lower intensity.

## Discussion

The main contribution of our study is to illustrate the utility of agent-based models to gain insight into fundamental dynamics driving patterns of active travel to school. We also show how the model can be used to identify the plausible impact of various policies or interventions. Our results indicate that to maximize the percent of children who walk to school the school locations should be evenly distributed over space and children should be assigned to the closest school. We also illustrate the beneficial impact of smaller catchment areas and higher population density. In the case of interventions to improve traffic safety, targeting a smaller area around the school with greater intensity may be more effective than targeting a larger area with less intensity.

### The impact of school location, catchment area definition, school size and population density

We used our model to illustrate how ABMs can help shed light on the impact of policies affecting school location and school catchment area which jointly determine the distribution of the distances to schools. The baseline scenario has the highest percentage of children walking to school because in this scenario, schools are distributed evenly across the city, and this minimizes the distances from households to schools across the whole sample. The percent of children walking to school is higher in scenario S3 than in scenario S1, because both scenarios allow the children to attend the school which is nearest to their household, but in S3, schools were located randomly within the zone while in S1, schools were located randomly within the whole city. Thus, on average the probability that schools are too close to each other (resulting in large distances for some children) is smaller in S3 than S1. Scenario S3 is also better than S2, because although the rules for the school location are the same in both scenarios, in S3 the catchment area is based on distance to the school resulting in a distribution of distance to school that is more skewed towards shorter distances. Walking levels in scenario S1 are very similar to S2. The implication is that the disadvantages in terms of ATS of the school location strategy for S1 (compared to S2) can be compensated by an advantageous school catchment policy that requires students to attend the closest school.

The school location and catchment polices in our scenarios are clearly unrealistic. However, a major advantage of applying ABMs to these questions is the ability to predict the joint impact of policies affecting multiple different domains (such as school location and catchment areas) in quantitative terms. The exploration of various more complicated “what if “scenarios can allow identification of the optimized school location and catchment area strategies [41] appropriate for different contexts, for example, across different spatial patterns of land use mix or in cities with varying spatial distributions of crime or traffic safety.

It is not surprising that with a fixed total number of households, a larger number of schools will increase the percent of children who engage in ATS. This is because more schools leads to smaller catchment areas and shorter distances to schools. This can also account for the diagonal pattern observed in Table 3: when the school size is constant (750 children each school), a higher population density means smaller catchment areas. An interesting insight arising from our model is that with a fixed number of schools (that is fixed school catchment areas), a higher population density also results in higher percent of children using ATS. This is because, when catchment areas are held constant, a higher population density will result in higher absolute number of children walking to school. This increase in walkers increases the traffic safety level which in turn results in more walking, leading to a reinforcing feedback loop.

Using the model, we provided a possible explanation of the mechanism by which high population density could increase ATS. Of course, this explanation is based on the assumptions encoded in our model including the assumption that the traffic level is only affected by the number of walkers which may be incorrect. For example, it is possible that higher population density will also result in higher absolute number of parents who drive their children to school which could have a detrimental effect of traffic safety. Nevertheless, our results illustrate the potential utility of ABMs, more specifically the ability of these models to identify dynamic mechanism and explain the synergistic effects of various features through modelling of simple feedbacks.

### Changing the traffic safety level

Our results show that strategies that target a smaller area around the school with greater intensity are more effective in increasing ATS than strategies that target a broader area with lower intensity. The implication of this finding is that investments to increase safety should begin in the areas closer to school and then extend to remote areas, in order to maximize the benefit. At least three different processes could account for this result: (1) increasing traffic safety close to the school with greater intensity results in a greater improvement because it directly and intensely influences children living shorter distances from school who are more likely to respond to changes in safety (children living further from the school will not be affected as much because distance becomes a barrier to walking regardless of safety). (2) increasing traffic safety closer to the school is beneficial to all children because all children using ATS must walk through the area around the school; (3) the strategy also has an important indirect effect because it changes the “social norms” regarding ATS within the school, which in turn affects the attitude towards walking for all children within the school regardless of whether they live in the area targeted for intervention. This means that children living out of the area targeted for safety improvements can benefit as well.

Among the three processes, the first two could be inferred through reasoning, but identification of the third process requires modelling. By including a feedback mechanism in the model through which each child’s attitude towards walking is affected by school social norms, we allow children to influence each other. Another example of a dynamic process in our model is the dynamic interaction among travel modes especially between walking and driving. Some of the traffic around the schools is contributed by the parents themselves [42]. When many parents drive their children, other parents may feel obliged to do the same to avoid traffic related risks to their children if they walk or cycle [43,44]. On the other hand, if more children walk or bicycle to school, the traffic around the school may be alleviated and the level of traffic safety may be increased, which may in turn encourage more children to join ATS. The explicit implementation of these dynamic processes in the model allows us to test intervention strategies that could trigger these kinds of feedback loops, such as an education campaign to increase the children’s attitude towards active travel. Modelling approaches can allow us to identify the existence of a “tipping point” beyond which the feedback triggers a trend in the system in the direction we desire. We can also investigate ways to maintain the desired tendency. The implication is that we should take advantage of these “self-reinforcing” feedbacks to encourage ATS and maximize the benefits from limited investment.

The cases presented in this paper are very simple illustrations, but already begin to shed light on some fundamental dynamics that may be operating. The use of the model to answer more specific research questions will require refinements. For example, bicycling and using a school bus (or other public transportation) may be added as additional travel modes. It is also possible to modify the model by allowing for greater heterogeneity across children and households. For example, marked variations in ATS among children by age, gender and income level have been reported [23]. These refinements may be especially important if the research questions are related to differences in the distribution of ATS across various populations. Another important improvement is to add more dynamic processes to the model. Currently, two dynamic processes were implemented for illustrative purposes. More dynamic interactions may be identified as important to ATS including for example, the interaction between the travel modes of various family members. If there is more than one child within a family enrolled in the same school, the probability of ATS may increase because children can walk together to be safe from traffic and crime [45]. Also, a parent who walks to his/her workplace may be more likely to chaperone a child who is walking if spatially convenient. In addition, the impact of variations across features of the built and social environment such as land use mix and density, safety from crime and weather could be explored.

A major challenge in developing these models is limited knowledge on the specific processes that are involved in decision-making regarding mode choices for travel to school. Another challenge pertains to making these models empirically grounded given that data to support some parameters may be unavailable or may be impossible to obtain. Despite these challenges, our examples illustrate the potentialities of ABMs to gain insights that cannot be obtained from observational data and to generate hypotheses that can be tested with other standard approaches including usual observational and experimental studies.

## Competing interests

The authors declare that they have no competing interests.

## Authors’ contributions

YY originated the study, designed and implemented the model, performed data analysis, and drafted the manuscript. AVD provided feedback on research questions and model design, helped to interpret the results and draft the manuscript, and provided financial support. Both authors read and approved the final manuscript.
